# Organising Pneumonia as an Initial Manifestation of Rheumatoid Arthritis: A Case Report and Review of Literature

**DOI:** 10.31138/mjr.34.1.110

**Published:** 2023-03-31

**Authors:** Nayan Patel Sureja, T Anuradha

**Affiliations:** 1 Department of Rheumatology and Clinical Immunology, Star Hospitals, Hyderabad, India,; 2 Department of Pulmonology, Star Hospitals, Hyderabad, India

**Keywords:** interstitial lung disease, organising pneumonia, rheumatoid arthritis

Dear Editor,

Interstitial lung disease (ILD) is a frequent extra-articular manifestation of rheumatoid arthritis (RA), which usually manifests several years after the onset of articular symptoms. However, less than 10% of RA patients have ILD as an initial manifestation.^1,2^ Organising pneumonia (OP) preceding articular manifestations of RA is extremely uncommon with only few cases reported.^1–8^

A 28-year-old female with three months of gestation, presented with dry cough for one year, and symmetrical inflammatory polyarthritis involving small and large joints of all the extremities for one month. Examination revealed multiple tender joints, and swollen left wrist joint. Fine inspiratory crepitations were heard on auscultation over both the lungs. Rest of the physical examination was normal.

On evaluation, complete blood counts, urine examination, creatine phosphokinase, renal and liver function tests were normal. Computed tomography imaging of the chest (performed five months prior to the present presentation) was suggestive of OP (**Figure 1**). C-reactive protein was elevated, rheumatoid factor (RF) was positive (20 times above normal limit), and anti-cyclic citrullinated peptide antibodies (ACPA) were negative.

**Figure 1.**
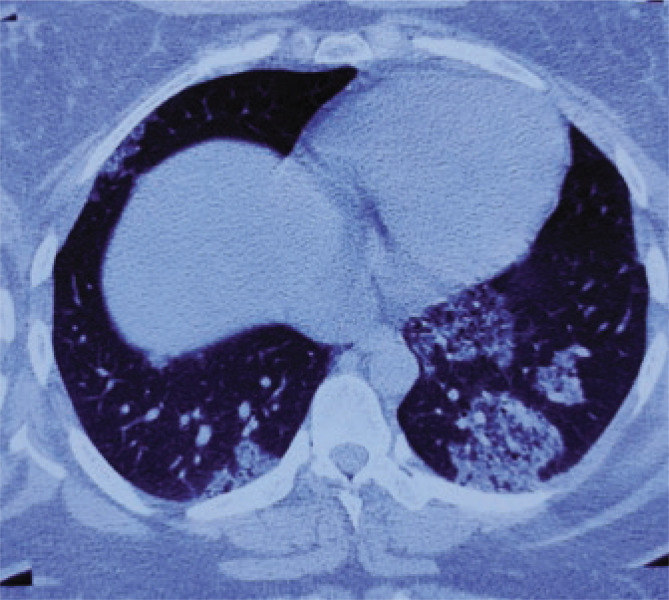
Organising pneumonia. Computed tomography of the chest showing multiple consolidations with central ground glassing (reverse halo sign), predominantly in the peripheral and subpleural locations in both the lungs.

Anti-nuclear antibodies by indirect immunofluorescence on Hep-2 cells at 1:100 titre showed fine speckled nuclear pattern. Line immunoassays for myositis specific/associated and anti-extractable nuclear antigen antibodies were negative. Anti-neutrophilic cytoplasmic antibodies were negative, and serum complement (C3, C4) levels were normal. Forced vital capacity on spirometry was 80%. Sputum did not yield any pathogenic organisms. Polymerase chain reaction for mycobacterium tuberculosis antigen in the sputum was negative. Lung biopsy was deferred, as the clinical and radiological characteristics of the lung lesions were consistent with OP. With a diagnosis of seropositive RA with ILD, patient received oral prednisolone 30 mg/day (with tapering of 5 mg every fortnightly), and sulfasalazine 2 gms/day. Within next few days, her pulmonary and articular symptoms subsided.

OP is a type of ILD, histologically characterized by buds of granulation tissue within the bronchioles and alveoli.^6^ It is called cryptogenic OP when the etiology remains uncertain, and secondary OP when associated with infections, drugs, and connective tissue diseases including RA.^9^ Compared with other types of ILD seen in RA, such as nonspecific interstitial pneumonia and usual interstitial pneumonia, OP is less common and has better prognosis.^8^ Occasionally OP may resolve spontaneously. However, most of the patients require glucocorticoids, which is the standard of care. Cyclophosphamide can be used in patients not responding to corticosteroids.^10^

PubMed search with terms “rheumatoid arthritis” AND (“organising pneumonia” OR “organizing pneumonia” OR “organising pneumonitis” OR “organizing pneumonitis” OR “BOOP” OR “interstitial lung disease”) identified 11 patients of RA with OP as an initial manifestation, published in English literature.^1–8^ Clinical characteristics of these patients including the present case are summarised in **Table 1**. The median age of these patients was 61.5 years (range: 28–86), with present case being the youngest, and 8/12 patients were female.

**Table 1.** Previously reported cases of rheumatoid arthritis with organising pneumonia as an initial manifestation.

**Author**	**Age**	**Sex**	**Arthritis onset after OP (months)**	**RF at onset of OP**	**RF at onset of arthritis**	**ACPA at onset of OP**	**ACPA at onset of arthritis**	**Treatment**
Kalinova et al.^3^	56	F	4	+	+	+	+	MP 40 mg
Hoshino et al.^4^	71	M	0.75	+	+	+	+	Pred 30 mg
Kinoshita et al.^1^	58	M	24	+	+	+	+	Pred 40 mg
Komiya et al.^2^	86	F	8	+	+	+	+	Pulse steroid
Cavallasca et al.^5^	65	M	6	−	+	NA	+	Pred 80 mg
Ippolito et al.^6^	68	F	0.5	NA	+	NA	NA	Pred 60 mg
Henriet et al.^7^	69	F	8	NA	+	NA	+	Pred 1 mg/kg
	33	F	3	NA	+	NA	+	Pred 40 mg
Mori et al.^8^	41	F	32	+	+	+	+	Pred 40 mg
	53	M	27	+	+	+	+	Pulse followed by Pred 50 mg
	65	F	4	+	+	+	+	Pulse followed by Pred 50 mg
This case	28	F	11	NA	+	NA	−	Pred 30 mg

F: Female; M: Male; MP: Methylprednisolone; Pred: Prednisolone; OP: Organising pneumonia; RA: Rheumatoid arthritis; RF: Rheumatoid factor; ACPA: Anti-cyclic citrullinated peptide antibodies.

At the onset of respiratory symptoms, RF and ACPA were positive in 7/8 and 7/7 patients respectively. Whereas at the onset of articular symptoms, RF was positive in all the patients, and 10/11 patients had ACPA. In the present case it is uncertain whether the antibodies were present at the onset of ILD or developed thereafter.

The present case along with previously published cases emphasises on the importance of testing for RF and ACPA, and a cautious follow-up for development of arthritis in patients with cryptogenic OP.

## References

[1] Kinoshita Y Sakamoto A Hidaka K. Organizing pneumonia preceding rheumatoid arthritis. Case Rep Pulmonol 2014;2014:758619. 24600522 10.1155/2014/758619 PMC3926251

[2] Komiya K Teramoto S Kurosaki Y Kashizaki F Kawashima M Masuda K Organizing pneumonia with a positive result for anti-CCP antibodies as the first clinical presentation of rheumatoid arthritis. Intern Med 2010;49:1605–7. 20686298 10.2169/internalmedicine.49.3609

[3] Kalinova D Kolarov Z Rashkov R. Organising pneumonia - the first manifestation of rheumatoid arthritis. Reumatologia 2017;55:314–7. 29491540 10.5114/reum.2017.72629 PMC5825970

[4] Hoshino C Satoh N Narita M Kikuchi A Inoue M. Organising pneumonia as the first manifestation of rheumatoid arthritis. BMJ Case Rep 2011;2011:bcr1120103558. 10.1136/bcr.11.2010.3558 PMC3070370 22699479

[5] Cavallasca JA Caubet M Helling CA Tate GA. Cryptogenic organizing pneumonia (COP), as presentation of rheumatoid arthritis. Rheumatol Int 2008;29:99–101. 18500458 10.1007/s00296-008-0618-4

[6] Ippolito JA Palmer L Spector S Kane PB Gorevic PD. Bronchiolitis obliterans organizing pneumonia and rheumatoid arthritis. Semin Arthritis Rheum 1993;23:70–8. 8235666 10.1016/s0049-0172(05)80027-7

[7] Henriet AC Diot E Marchand-Adam S de Muret A Favelle O Crestani B Organising pneumonia can be the inaugural manifestation in connective tissue diseases, including Sjogren’s syndrome. Eur Respir Rev 2010;19:161–3. 20956186 10.1183/09059180.00002410 PMC9682575

[8] Mori S Koga Y Sugimoto M. Organizing Pneumonia in Rheumatoid Arthritis Patients: A Case-Based Review. Clin Med Insights Circ Respir Pulm Med 2015;9:69–80. 26543387 10.4137/CCRPM.S23327 PMC4624096

[9] Drakopanagiotakis F Polychronopoulos V Judson MA. Organizing pneumonia. Am J Med Sci 2008;335:34–9. 18195581 10.1097/MAJ.0b013e31815d829d

[10] Raghu G Meyer KC. Cryptogenic organising pneumonia: current understanding of an enigmatic lung disease. Eur Respir Rev 2021;30:210094. 34407978 10.1183/16000617.0094-2021 PMC9488952

